# A novel plot for the early alert of epidemic growth using regional targets: the doubling plot: Fabrizio Carinci

**DOI:** 10.1093/eurpub/ckac129.344

**Published:** 2022-10-25

**Authors:** F Carinci, G Veltro, L Rocchi, D Mipatrini, D Mantoan, G Siccardi

**Affiliations:** 1 Information and Communication Technologies, Italian National Agency for Regional Health Services, Rome, Italy; 2 Department of Statistical Sciences, University of Bologna, Bologna, Italy; 3 General Directorate of Health Prevention, Ministry of Health, Rome, Italy

## Abstract

**Background:**

During the pandemic, restrictions set by the Italian Government were primarily based on the regional level of key parameters including hospitalization and incidence rates. We aimed to build a specific plot to monitor trends and trigger early alerts, with daily updates publicly available on a National Portal.

**Methods:**

A multidisciplinary team conceived and implemented a new composite plot, developing ad hoc R scripts on top of a specialised database, built in collaboration with the Ministry of Health. We calculated the doubling time Td as log(2)/log(1+r/100), where r is the daily change of target parameters and Td ranges between (0,+-infinity), and not determined for constant or missing values. We calculated Td daily, as either doubling (growth) or halving (decrease) time. To visualize trends, we assembled two different types of graphs: a bivariate plot showing the path of each point (Td, target parameter) over time, and a line plot of Td over time. The Y axis was inverted for doubling times, as lower Td indicate higher alert in this case. The two graphs were arranged in lines, using cutoffs for excessive high values for doubling times and low values for halving times. A third line was included to display trends of the target parameter over time.

**Results:**

The plot was successfully realized and published on the Portal for all regions in February 2021 (https://www.agenas.gov.it/covid19/web/index.php?r=english%2Fdoubling&q=ITA&t=0). Since July 2021, we used the doubling plot to monitor the three main parameters adopted to set restrictions for Covid-19: a) occupancy rates in intensive care; b) occupancy rates in medical wards; c) weekly incidence rates. The plot highlighted growth trends and early alerts, particularly in the initial phases of growth.

**Conclusions:**

The doubling plot can provide useful information to trigger early responses for pandemic control in decentralised governance. R code is available open source from AGENAS for free use.

**Key messages:**

• The doubling plot was conceived and implemented on a National Portal to trigger early alerts of Covid-19 progression in Italian Regions and Autonomous Provinces.

• The plot could be rapidly adapted to legislative parameters and can be useful in different situations to monitor epidemic growth and support public health policies.

